# Qualitative insights into promotion of pharmaceutical products in Bangladesh: how ethical are the practices?

**DOI:** 10.1186/s12910-015-0075-z

**Published:** 2015-12-01

**Authors:** Mahrukh Mohiuddin, Sabina Faiz Rashid, Mofijul Islam Shuvro, Nahitun Nahar, Syed Masud Ahmed

**Affiliations:** University Press Limited, Dhaka, Bangladesh; James P Grant School of Public Health (JPGSPH), BRAC University, Dhaka, Bangladesh; Mainstreaming Nutrition in Bangladesh, Dhaka, Bangladesh; Centre for Equity and Health Systems, icddr,b and Centre of Excellence for Universal Health Coverage, icddr,b and JPGSPH, icddr,b and JPGSPH, BRAC University, 5th Floor(Level-6), icddrb Building, 68 ShahidTajuddin Ahmed Sharani, Mohakhali, Dhaka, 1212 Bangladesh

**Keywords:** Medical representatives, Pharmaceutical marketing, Pharmaceutical promotional gifts, Code of Pharmaceutical Marketing Practices, Bangladesh

## Abstract

**Background:**

The pharmaceutical market in Bangladesh is highly concentrated (top ten control around 70 % of the market). Due to high competition aggressive marketing strategies are adopted for greater market share, which sometimes cross limit. There is lack of data on this aspect in Bangladesh. This exploratory study aimed to fill this gap by investigating current promotional practices of the pharmaceutical companies including the role of their medical representatives (MR).

**Methods:**

This qualitative study was conducted as part of a larger study to explore the status of governance in health sector in 2009. Data were collected from Dhaka, Chittagong and Bogra districts through in-depth interview (healthcare providers and MRs), observation (physician-MR interaction), and round table discussion (chief executives and top management of the pharmaceutical companies).

**Results:**

Findings reveal a highly structured system geared to generate prescriptions and ensure market share instituted by the pharmaceuticals. A comprehensive training curriculum for the MRs prepares the newly recruited science graduates for generating enough prescriptions by catering to the identified needs and demands of the physicians expressed or otherwise, and thus grab higher market-share for the companies they represent. Approaches such as inducements, persuasion, emotional blackmail, serving family members, etc. are used. The type, quantity and quality of inducements offered to the physicians depend upon his/her capacity to produce prescriptions. The popular physicians are cultivated meticulously by the MRs to establish brand loyalty and fulfill individual and company targets. The physicians, willingly or unwillingly, become part of the system with few exceptions. Neither the regulatory authority nor the professional or consumer rights bodies has any role to control or ractify the process.

**Conclusions:**

The aggressive marketing of the pharmaceutical companies compel their MRs, programmed to maximize market share, to adopt unethical means if and when necessary. When medicines are prescribed and dispensed more for financial interests than for needs of the patients, it reflects system’s failed ability to hold individuals and entities accountable for adhering to basic professional ethics, code of conduct, and statutory laws.

## Background

Promotion of pharmaceutical products involves “all informational and persuasive activities by manufacturers and distributors, the effect of which is to induce the prescription, supply, purchase and/or use of medicinal drugs” [1]. Pharmaceutical industries worldwide are heavily involved in aggressive promotion of medical products [2, 3]. Interactions between physicians and pharmaceutical industries begin as early as medical school days and continue well into professional life [4]. This interaction has increased significantly over the last few decades. The pharmaceutical industries spend between 15 and 25 % of their total budget on promotional activities, which is even higher in the third world countries [5]. The aggressive pharmaceutical promotion can pose an ethical threat to professionalism because such activities may influence prescribing behavior of physicians without benefiting the patients [6]. This type of interactions between pharmaceutical industries and physicians were found to have negative outcomes that compromise patient’s best interests [7, 8].

One of the tools commonly used by the pharmaceutical companies is offering gifts (from stationeries to household items to overseas trips to attend conferences, etc.) to motivate physicians to write prescriptions [9–12]. Acceptance of these gifts, especially the expensive ones, obliges them to return favour by changing established prescription norms and increasing sales [13]. Evidence shows that, though the pharmaceutical companies initiate the unethical marketing practice, physicians are responsible for its continuation [14]. Conflicts of interests among the stakeholders prevent proper implementation of different regulations and guidelines formulated by the government for ensuring ethical practices [15]. This problem is severe especially in low and middle-income countries, where supervision and regulation of the pharmaceutical industries are weak.

Bangladesh became the first low-income country to develop an indigenous pharmaceutical industry [16, 17]. Now it claims a market share of more than 75 % of total drug sales compared to 25 % before the National Drug Policy was enacted in 1982. The pharmaceutical market in Bangladesh is highly concentrated and limited to a few big companies. The top ten companies have 68 % of the market share and the top 20 have 78 %. Currently, there are 265 allopathic drug manufacturing companies in Bangladesh, of which 30 are considered large-scale units that dominate the market [18]. Due to high competition in the industry, aggressive marketing strategies have been adopted by the different companies. In this respect, promotion has become a useful tool to fight competition. Like other countries, health professionals in Bangladesh are also targeted by companies mainly through medical representatives (MR) for promotion of medicinal products. One to one visits from the MRs have been proven to be the most effective way to promote drugs to physicians, because they can identify the main motivators and decision-making styles of the person they are selling to and adapt to their approach accordingly [19]. Visits from MRs are usually associated with providing gifts, free samples, and advertising campaigns.

There is a lack of data on MRs’ unethical practices for promotion of pharmaceutical products in Bangladesh, albeit the presence of a Code of Pharmaceutical Marketing Practices promulgated in 1994. This exploratory study aimed to fill this data gap by investigating current practices of the pharmaceutical companies in this regard including the role of pharmaceutical sales representativesin influencing prescribing practices of the physicians. Findings from this study are expected to help develop a drug promotion practice which is transparent and accountable and regulated by relevant authority.

## Methods

This study was conducted as part of *Bangladesh Health Watch* exercise to explore the status of governance in health sector in 2009 [20]. The information sought being of sensitive nature, we adopted a qualitative approach and a purposive sampling strategy. Respondents were recruited from Chittagong and Bogra districts besides the capital Dhaka to explore variations in promotional practices and regulations, if any. The sites outside Dhaka were selected based on consultation with the Technical Advisor of the study team – Bogra being an important drug distribution depo tin the northern part of the country and Chittagong being a representative of the south/south-eastern part. Data were collected through in-depth interviews with the healthcare providers and MRs, real-time observation of a sample of the physician-MR interactions, and round table discussions with chief executives and members of senior management of the pharmaceutical companies (Table 1). Due to time and resource constraints purposive sampling was done to select the respondents.

**Table 1:** Respondents in the study

Data collection	Sample	
1. Observation (interactions between physicians and MRs)	16 observations	
a. Public & private institutes	4 Public Healthcare Institutions (medical college hospitals)	
	2 private healthcare facilities(doctor’s chamber/private clinic)	
b. Pharmacies/drug shops	10 Drug shops from each site	
2. In-depth Interview		
a. Authorities	5 Authorities(DGDA, BMDC, BIRDEM, Ibrahim Medical College, CAB)	
b. Doctors	14 Doctors	
		• 3 upazila health complexes (1 from each site) • 3 district hospitals (1 from each site) • 3 divisional medical college hospitals (1 from each site) • 5 private healthcare facilities(doctors chamber/private clinic, 1 from each division
c. *Pallichikitshoks* (PCs, village doctors)	8 *Pallichikitshoks* (PCs, village doctors)	
d. MRs	11 MRs (Interviews wereconducted with MRs of variouspharmaceutical companies, both local and multinational)	
3. Round-table discussion	Participants from pharmaceutical companies:	
	13 participants (CEO, MD, ED, sales manager/senior marketing manager/Director Marketing, manager medical affairs etc.) from 10 renowned pharmaceutical companies	
	Other Participants:	
	2 academicians (asst. professor of pharmacy and PhD student, pharmaceutical law and marketing)	

### In-depth interviews

The in-depth interviews were sought to elicit information on topics like role of MRs in detailing products to healthcare providers, securing information on personal preferences and life-styles of the providers, maintenance of database, quantity and quality of gifts offered, personalized services provided, and adopting different other promotional approaches to fulfill sales targets. Eleven interviews were conducted with MRs of various pharmaceutical companies, both local and multinational; also interviewed were 14 doctors, eight *pallichikitshoks* (village doctors), and five representatives from DGDA (Directorate General of Drug Administration), BMDC (Bangladesh Medical & Dental Council), BIRDEM (Bangladesh Institute of Research & Rehabilitation in Diabetes, Endocrine and Metabolic Disorders), Ibrahim Medical College, and CAB (Consumer Association of Bangladesh) (Table 1). A guideline was prepared after review of the literature and discussion with technical adviser of the study. Each interview lasted for a maximum of 1 h.

### Observation

To cross check the data obtained from the in-depth interviews, realtime observations of the provider-MR interactions were made in 16 sites from the three districts, of which six were healthcare institutions (two private and four public) and ten were drug retail outlets. In these observations issues such as technical content of the interaction, persuasion and compliance, nature and amount of gifts offered including free drug samples, discussion over personal issues, etc. were noted. A structured form was used for this purpose. Observations were done for the full length of interactions which lasted for 10 to 20 min on average.

### Round-table discussion

Finally, a round-table discussion was held to know industry perspectives on existing regulations for promotion of pharmaceutical products and their role in promoting more transparent and uniform practices. Chief and senior executives (mostly from marketing departments) of 11 local and multinational pharmaceutical companies attended this discussion (Table 1).

#### Analysis

**Fig. 1:**
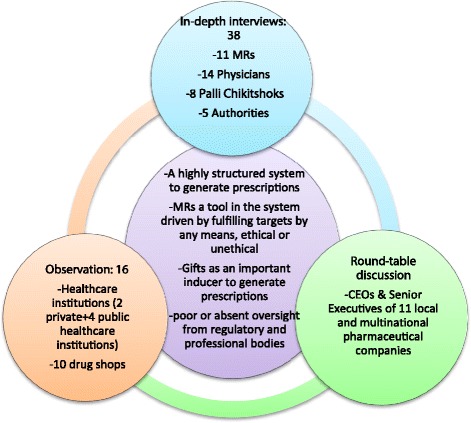
Triangulation of data for arriving at core themes of the findings

Content analysis was done of the data from in-depth interview with consolidation of sub-themes and themes. Triangulation of data was done by eliciting information on a particular issuefrom different sources e.g., data on gifts offered (Fig. 1).

#### Ethical approval

The study proposal passed through the ethical review board of the James P. Grant School of Public Health at BRAC University. No invasive procedure was done. Informed verbal consent was obtained from the respondents before conducting interview or documenting physician-MR interaction. Anonymity of the respondents was maintained at all stages.

#### Availability of data and materials

The comprehensive transcribed qualitative data are available with the authors and archived in the repository of the JPG School of Public Health at BRAC University.

## Results

The qualitative findings obtained from in-depth interview, healthcare provider-MR interaction and round-table discussion are summarized below.

### Medical Representatives (MR): the strategic link between pharmaceutical companies and physicians

The large pharmaceutical market was principally driven by the prescriptions of physicians. As such, the success of the pharmaceutical companies depended on the effectiveness and efficiency of their MRs. They were mainly science graduates, recruited through a competitive process. After recruitment, they went through a structured training programme which besides technical details on relevant products, also included how to observe and assess doctors’ personalities and preferences. Thus, they gathered doctors’ personal information such as family and lifestyle details, hobby, personal interests, etc. which is termed as “history taking” by the MRs.

Categorization of doctors based on their ‘history’ and how to approach them was also something that the MRs were trained on. For example, one of the well-known multinational companies categorized doctors (whom they refer to as customers) according to who values what (descriptions according to MR interview):

- Directing type: Doctors who have strong values and maintain their dignity. They only prefer to have information related to drug and not to talk about anything else and have no interest in other benefits.
- Thinking type: Quiet in nature, they take time to think before making any decision. They ask for reference/evidence on the promotional briefs. They usually prescribe the drugs only when they are convinced.
- Affiliating type: These doctors welcome the MRs, they like to listen to them and also express their opinions. *“It’s easier to work with this type of doctors,” said an MR.*
- Expressing type: These doctors often dominate the conversation while talking to the MRs, they hardly listen carefully to what the MRs say and usually do not prescribe the products on offer. *“They express their demands quite frankly.”*

This is only one example of how MRs were oriented to face real life dealings with their customers. This orientation of the MRs to meet their customers was used to fulfill doctors’ demands and ultimately persuade them into writing prescriptions as a favour in return (a ‘give and take’ policy, according to one MR).

### Dynamics of MR visits: blurring professional boundaries

The MR’s were encouraged to interact with physicians at personal level which is also appreciated by the physicians. Paying regular visits to physicians was one strategic way of maintaining this relationship, and sometimes there was a ‘blurring of professional boundaries’ when it came to this relationship. To illustrate:

*“Sometimes, in one visit, the doctor becomes convinced…sometimes, it take four to five visits…if the gap is long between two visits, then the doctor forgets about the drug, so we have to visit frequently to highlight our products…” –*One MR

A medicine specialist’s comment on the same issue supported the MR’s claim:

*“I automatically develop a soft corner for the MRs who visit me regularly. Then I try to prescribe 2–3 of his products. I think most of the doctors feel the same way.”–* A specialist doctor

Not all doctors were visited by the MRsin same frequency. Doctors with high patient load were always preferred by the MRs. According to the MRs, they categorized doctors based on their ‘potentiality’, determined by the number of patients visiting a doctor on any given day which is a function of prescriptions that can be generated. Besides patient load, ‘loyalty’ of the doctors towards a company (i.e., inclination to prescribe drugs of a particular company) was also an important factor for determining the frequency of MR’s visit. During busy hours, MRs would drop in to exchange social greetings with the doctors as an effective brand reminder which could result in a prescription soon after:

*“In institutions, the doctors start prescribing drugs from the morning but we are allowed to visit them after 11 AM when the doctors are almost done with their duty hours. So, even though I cannot visit him, I go to him every day and give him a salaam so that he remembers my brands” –* An MR

Our observations revealed that the MRs of different companies preferred to visit physicians early in the morning:

*“… if we can visit doctors in the early morning, he will have more opportunities to prescribe our brand which is more beneficial for us”–* An MR

Findings also reveal that MRs prefer public institutions to private more since doctors in the former gave them more time and allowed them to visit while the doctors were attending patients. Patient load being higher in these facilities, the probability of gaining prescription share was also higher.

*“Working in public sector health facilities is much easier… we can enter there anytime and work, even when the doctor is attending patients. But in private hospitals and clinics, we have to strictly follow their schedule…there are many restrictions! Usually rich people come to these places and they also don’t want to see us in the hospitals…”–*An MR

The MRs spent the second half of their working days in the chambers of private practitioners. All the MRs in in-depth interviews said that they liked to interact with the physicians during their private practice and nurture the relationship. One MR said,

*“In terms of visit, private chamber is more preferable to us. Because, in institutions sometimes we have to visit many doctors at a time and we have to ensure that all the doctors get equal amount of samples or gifts. But in private practice doctors sit in separate room…so they are easy to deal with and we can motivate them to prescribe our drugs.”–* An MR

However, as in most of the times the relationship between an MR and a physician is one of ‘give and take’, and it needs constant nurturing.

### Achieving targets: monitoring prescriptions

‘Target’ is very important for the MRs; each pharmaceutical company had an individual target (monthly, quarterly, annually) for its MRs. They received an increment or incentive based on targets achieved, which varied company to company. Thus, at the end of each quarter, MRs are under pressure to meet these targets by any means. Reportedly doctors, either out of sympathy or out of exasperation, ended up in prescribing some drugs under constant pressure from the MRs, especially around the end of quarters. To illustrate:

*“MRs are always anxious about meeting their targets… there are several MRs with whom I have good relationship. One of those boys came to me the other day and requested, ‘Sir, I need your help in fulfilling my target. If I can make it this time, I’ll be able to go to China.”– A physician*

While doctors succumbing to the persuasion of the MRs were common phenomena, there are exceptions where unwavering determination of the doctor sent a different message to the representatives:

*“… there have been occasions when MRs have appeared with blank cheques. I told them, ‘Do not spoil the doctors like this. They are also humans and they may also get temptated, so don’t do this to us.” –* A doctor

To fulfill the all ‘pervasive’ targets, several strategies were taken by the pharmaceutical companies. One of these was monitoring of prescriptions of senior professors and very successful private practitioners. According to the MRs, they were always tracking doctors and providing latest information to keep the companies’ database up to date:

*“When we visit a doctor we check whether the doctor who agreed to prescribe our product actually did so,…sometimes our company buys prescription survey data from other companies. We usually check the prescription from the patient…if our product is not prescribed we keep reminding them through frequent visits…”–* An MR

This prescription monitoring by the MRs served other purposes as well:

“…*by monitoring prescriptions, we can identify prescribing habits of a doctor. First, we can identify which drugs a doctor usually likes, so that we can promote those to increase our sale. Not only that, if a doctor has any special relation with a company, we can identify that by seeing most of the products of that company in the prescription and can form our strategy to [make them switch their preference].” -*MR

The study also revealed that the top pharmaceutical companies buy the prescription database from third parties such as Intercontinental Marketing Services (IMS) at a high cost. From this prescription database, they get the region-specific list for brand preference of each doctor which helps customize strategies appropriate to the prescribing patterns.

### Gifts, gifts, gifts: from a note pad and pen, to an overseas trip for work or pleasure!

The most commonly used promotional materials offered by the pharmaceuticals are literature on drugs, journals, writing pads, pens and sample drugs. But there was an increasing tendency of the pharmaceutical companies to provide almost everything as gifts to the doctors:

*“Pharmaceutical companies provide everything except kaacha bazaar. Even during the last Eid they provided perfumed rice (polao er chal), sugar, vermicelli (semai), etc. So, it’s not easy to say what they don’t offer us.”–*A doctor

The gifts included items for both professional and personal use. The professional items include sponsorship for attending medical conference and seminar; medical equipment and books; items for doctors’ waiting area (chairs, water filter, TV, etc.), personalized visiting card, prescription pads & prescription folders; and cash for products prescription. Items for personal use include costs of air ticket and hotel accommodation for pleasure trips with family and friends; decoration for home; exclusive gifts such as home, flat, car, etc. Other gifts include food items, mobile recharge cards, Internet modem, cash or sponsorship for personal programmes such as wedding, birthday, naming ceremony (*Akika*), etc.

The pharmaceutical companies used two approaches for offering gifts. In proactive approach, some inexpensive gifts were given to the doctors each month along with free samples of drugs during regular promotional visits of the MRs. Brand names were usually inscribed on these gifts so that it worked as reminders for the doctors. The other approach involved offering inducements based on doctors’ demands, i.e. “whatever s/he wants as gifts.” This culture of inducement has even been extended to the family members of doctors, especially the younger members. As illustrated by two MRs:

*“Sometimes doctors want to take their family members with them to the foreign trip and we sponsor them too. Besides sponsoring seminar and workshops, we also give doctors different kind of electronic and household items. We give every possible item they ask for…”–*An MR

*“Some doctors demand computer, TV, fridge, AC, mobile phone, laptop, etc. Mobile bill, electric bill, attendant bill, driver’s salary, paper bill, etc. - for everything they have contract with companies…” –*An MR

This is also supported by a doctor who recalled:

*“Dhaka-Singapore air tickets, cars, houses, and even family or household items – these are the things that doctors get. … Doctor X has bought a plot in Dhanmondi. Lots of people are becoming owners of cars and houses in this way. You can even call it a competition these days [of who receives more in terms of gifts].”–*A doctor

The changing pattern of promotional activitieshad an impact on the prescription habits of the doctors which was acknowledged by a doctor:

*“Promotional activities influence our prescription habit. I think that the doctors realize this but they stilldo it. When a doctor accepts a gift from a company, he knows why he is offered that….a certain obligation develops towards that company…”*

But there are exceptions too. A professor of a private medical college explained:

*“…doctors are frustrated of this corrupt system and the way it compels them to become a part of it.”*

The study reveals that doctors generally are not interested in receiving product literature from MRs, rather they are more interested in drug samples that MRs bring as promotional items. It is a common complaint that a substantial proportion of the drug samples for the physicians ultimately land in the retail drug outlets. Here are two quotes from MRs:

*“…we usually place the samples on the table (of the doctor)…it’s not right to sell these. But, we cannot question the doctors about this, neither the company takes any action in this regard”. –*An MR

*“Pharmaceutical promotion has now reached the stage of bribing. In earlier days doctors were given drug samples for their poor patients. ‘Not for sale’ was printed on the packets of the drugs and the doctors gave it to their patients. But nowadays, doctors are selling the drugs…they have taken their profession as a business”.–* An MR

### Market segmentation

The MRs of large and medium scale companies primarily target doctors to promote their products while the MRs of smaller companies usually target village doctors and the likes to sell their products. MRs agreed unanimously that they spent more time in detailing to village doctors rather than MBBS doctors, because village doctors were less knowledgeable about drugs and received MR visits more enthusiastically. Village doctors also expressed an interest in maintaining friendly relationship with the MRs and often bought drugs only from MRs who were “friends” with them.

### Awareness about Code of Pharmaceutical Marketing Practices (CPMP)

The MRs were unaware about the existing code on drug promotion and marketing practices. Many doctors also mentioned that they were unaware about the code:

*“I don’t have much idea about the CPMP. It is not clear to me which practices are considered unethical in our country. This needs to be clearly communicated to the doctors and also to the pharmaceutical companies. The Department of Drug Administration should then take an active role in preventing these unethical practices. I don’t think they are playing any role in this regard at present.” –* A doctor

The MRs perceived an ‘ethical doctor’ as one who is loyal to one particular company and an ‘unethical doctor’ is one who maintained contracts with multiple companies.

### Perspectives of the senior executives of the pharmaceutical companies

According to the executives of the pharmaceutical companies, the Code is voluntary. For their own interest, they try to abide by it. They said that they took prior approval from the authority (DGDA) for the literatures (including other promotional materials) before presenting them to doctors. The MRs are deployed only to detail the literature to the doctors. They claimed that the doctors are too busy to go through the literature on their own and most of the literatures landed in the trash. They argued that the companies don’t give unreasonable targets to the MRs as this would lead them to resort to unscrupulous means.

The top executives of the pharmaceutical companies also contended that inadequate capacity of the DGDA (both technical and human resource capacity) to monitor and regulate the market was responsible for unethical promotional practices. However, as the market is very competitive, they emphasized on the need for self-regulation, which according to them, is followed for the benefits of the industry. The executives also pointed out that the unregistered/unregulated drug retail outlets (drug shops) are the places where unethical promotional practices are rampant and attention should be directed there to put a check to these practices. They also reiterated the necessity for an official schedule of Over-the-Counter drugs to check unethical practices by the informal healthcare providers.

## Discussion

This study was done as part of a larger study by *Bangladesh Health Watch* 2009 which explored issues related to governance in the health sector including the pharmaceutical sector [20]. It aimed to investigate the extent of ethical procedures followed in the marketing practices of the pharmaceutical companies and the role of their MRs in this process. Findings reveal a structured and evidence-based drug promotion strategy instituted by the pharmaceutical companies which frequently violates ethics. The MRs are trained to act as a tool to achieve targeted market share through building personal relationship with the physicians and catering to their needs and demands, expressed or otherwise, and fulfill their personal targets as part of gaining higher market-share for the companies they represent. The physicians, willingly or unwillingly, become a pawn of the system with few exceptions. These are discussed with context in the following paragraphs.

### A highly structured system to generate prescriptions and ensure market share

As in other south Asian countries [2, 8], the pharmaceutical companies in Bangladesh have developed a comprehensive, evidence-based system (through ‘history’ taking of physicians and monitoring of prescriptions) in order to guarantee that enough prescriptions are generated to ensure targeted market share. This system is based on the army of the ‘MRs’ since they are found to be the best tool of promotion for a pharmaceutical company [21–23]. An evidence-based training curriculum prepares the newly recruited science graduates to learn how to study and assess the likes and dislikes, inclinations, and financial needs and demands of their ‘customers’, i.e. the physicians, besides technical knowledge on relevant products. They keep track of these and are ready to serve them when needed.

A variety of approaches are used such as giving gifts (from minor professional items to costly personal use items), persuasion, emotional blackmailing, support to the family members, and support in times of personal emergencies, etc. In doing these, they pay little heed to the laws or ethics. The MRs try to develop an intimate relationship with the physicians which they exploit to achieve their ‘targets’ [24]. The success of the MR’s career also depends on fulfilling the targets on time, which may result in stress with health consequences [25]. Thus, MRs appear to be pawns in a system, pre-programmed to achieve a certain target, whatever the efforts and costs. In the ‘unholy’ alliance of the pharmaceutical companies, MRs and the physicians, they are the least powerful [26].

### Offering gifts: crossing the ethical boundaries

The amount, quantity and quality of gifts offered to the physician depend upon his capacity of generating prescriptions. Those with high popularity are cultivated meticulously by the MRs to establish brand loyalty and generate enough prescriptions to fulfill both individual and company targets. However, the fresh graduates are nurtured from the very beginning to ‘co-opt’ them into the system. It is interesting that personal use items, including cash, accounts for a large portion of the gifts on offer which transcend all ethical boundaries. Free ‘sample’ drugs are also a problem [27, 28] especially when these land in retail outlets. The willing and sometimes ‘helpless’ surrender of the physicians to the aggressive marketing techniques of the pharmaceuticals (through the MRs) is mainly responsible for sustaining and nourishing the system [29]. This is compounded by the absence of any oversight from either the professional bodies or the regulatory bodies to discourage or curb this. Interestingly, the most preferred information source perceived by doctors such as educational programmes like conferences and seminars [30] is given hardly any importance in Bangladesh.

### The regulatory environment

The MRs are not aware about the existing code of marketing practices for pharmaceuticals, neither the majority of the doctors. There are also no efforts on the part of the regulators (DGDA) to disseminate this information. One reason frequently cited by DGDA is the shortage of their manpower to match the activities of the increasing number of pharmaceutical companies. Also, complete absence of the physicians’ professional bodies like Bangladesh Medical & Dental Association in overseeing the professional conducts of its members has given a free reign to the pharmaceuticals in doing whatever they deem necessary to boost sales of their products.

## Conclusions

Pharmaceutical companies expand market and maintain high profit through aggressive promotion of their products in Bangladesh without any hindrance. These promotional activities compel the MRs, programmed to maximize sale of products, to adopt unethical means if and when necessary. When medicines are prescribed and dispensed more for financial interests of the prescribers and dispensers than for needs of the patients, it reflects failure of the system’s ability to hold individuals and entities accountable for adhering to basic medical ethics, standard procedures, norms, laws and regulations. Both the regulatory and professional bodies have a role to play. Without increasing transparency and accountability in drug promotion the issues of public health and consumer rights will remain tangential.

## Abbreviations

BMDC: Bangladesh Medical & Dental Council
BIRDEM: Bangladesh Institute of Research & Rehabilitationin Diabetes, Endocrine And Metabolic Disorders
CPMP: Code of Pharmaceutical Marketing Practices
CAB: Consumer Association of Bangladesh
DGDA: Directorate General of Drug Administration
ICDDR, B: International Centre for Diarrhoeal Disease Research, Bangladesh
IMS: Intercontinental Marketing Services
JPGSPH: James P Grant School of Public Health
MR: Medical Representative
NDP: National Drug Policy
